# Introduction to the themed collection on XNA xeno-nucleic acids

**DOI:** 10.1039/d2cb90036j

**Published:** 2022-10-11

**Authors:** Dennis Bong, Philipp Holliger, Chaoyong Yang

**Affiliations:** Department of Chemistry and Biochemistry, Ohio State University 285 CBEC Building, 151 W Woodruff Ave Columbus Ohio 43210 USA bong.6@osu.edu; MRC Laboratory of Molecular Biology, Francis Crick Avenue, Cambridge Biomedical Campus Cambridge CB2 0QH UK ph1@mrc-lmb.cam.ac.uk; Department of Chemical Biology, Xiamen University Xiamen 361005 China

## Abstract

Dennis Bong, Philip Holliger, and Chaoyong Yang introduce the *RSC Chemical Biology* themed collection on XNA xeno-nucleic acids.
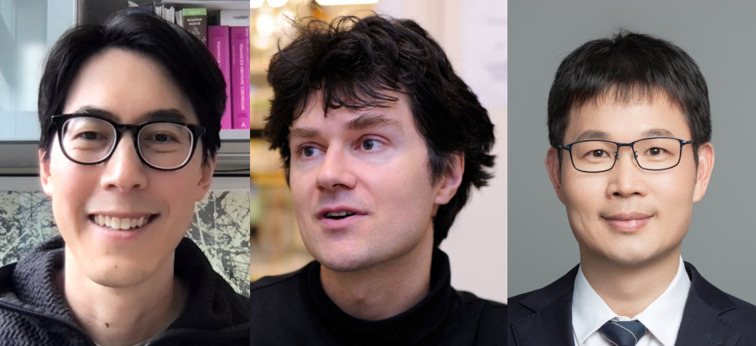

The concept of xeno-nucleic acids (XNAs) was first proposed in 2009 in a theoretical paper, referring to additional types of nucleic acids, whose sugar moieties would differ from those in DNA and RNA. However, with the rising popularity of XNAs, the definition of XNAs has been extended to unnatural nucleic acids with chemically modified sugar, nucleobase, or phosphate moieties that are distinct from those found in DNA and RNA. The discovery and engineering of both polymerases and reverse transcriptases to synthesize, replicate and evolve a diverse range of XNAs has attracted significant attention and has enabled the discovery of XNA ligands (aptamers) and XNA catalysts (XNAzymes) as well as the synthesis of XNA nanostructures with potential as novel therapeutics. The field of XNAs continues to grow rapidly towards realizing the potential of XNAs in biotechnology and molecular medicine. This themed issue unites a collection of articles attesting to the rapid progress in the field.

One of the key advantages of XNAs is their generally enhanced resistance to nuclease degradation. This biostability, the affinity and specificity towards a target, and the general lack of immunogenicity of modified nucleic acids are critical for their potential application as therapeutics. Modified sugar moieties such as 2′-modified analogs, conformationally locked analogs, and threose-replaced analogs in particular contribute to the increased biological stability of XNAs against enzymatic degradation. Replacing the phosphodiester linkages with charge-neutral backbones including peptide-like backbones and triazole-linked backbones offers further opportunities to tune the stability, conformation and physicochemical properties of XNAs and enhance the affinity to their targets. In this themed issue, Suparpprom and Vilaivan discuss the development of conformationally constrained peptide nucleic acids (PNAs) and provide perspectives on the rational design of PNAs with improved performance for further applications (https://doi.org/10.1039/D2CB00017B).

As synthetic genetic polymers, XNAs have the potential for Darwinian evolution, yielding specific XNAs with defined structures and complex functions. For example, by engineering the sugar, nucleobase, or phosphate moieties, XNA-based aptamers with superior performance can be evolved by systematic evolution of ligands by exponential enrichment (SELEX). Furthermore, SELEX using fully modified XNA libraries represents the most powerful tool for XNA-based aptamer evolution. However, natural DNA and RNA polymerases are inefficient in mediating transcription and reverse transcription between DNA and XNAs. Substantive effort in polymerase engineering has been applied to address this shortcoming by selective mutation and directed evolution of native polymerases with the goal of obtaining new polymerases with high efficiency and fidelity for XNA synthesis. Herein, Taylor and colleagues use a previously established XNA polymerase to generate XNA libraries of 2′OMe and locked nucleotides and demonstrate that the XNA libraries can be efficiently synthesized and replicated with high accuracy (https://doi.org/10.1039/D2CB00035K).

Validated XNA polymerases thus enable both storage and retrieval of genetic information from XNA scaffolds. Moreover, it is possible to create semi-synthetic organisms by adding XNAs into the genetic alphabet as new base pairs to yield unnatural codon-embedded mRNAs coding for non-proteinogenic amino acids. Chen and colleagues provide a comprehensive review on this approach to artificial expansion of the central dogma using XNAs and engineered polymerases (https://doi.org/10.1039/D2CB00116K). Indeed, fully XNA-compatible polymerases with native-like function are needed to drive further expansion of central dogma and enable the creation of synthetic organisms with tailored function.

Though this themed issue in *RSC Chemical Biology* is but a small sampling of a rich research area, it nevertheless highlights recent progress in XNAs in chemical and synthetic biology, encompassing fundamental investigation of biological processes, new chemical and biological technologies and translational research. As guest editors, we are excited by and very grateful for the diverse contributions from all authors, that together illustrate the interdisciplinary aspect of this journal and underscore the value of this published forum to the broad chemical biology community. We hope that these articles will inform and inspire researchers in chemistry, biology, physics, and beyond to further advance the development of XNA technology.

## Supplementary Material

